# Clinical and mutational spectrum of Colombian patients with Pelizaeus Merzbacher Disease

**DOI:** 10.25100/cm.v49i2.2522

**Published:** 2018-06-30

**Authors:** Harvy Mauricio Velasco Parra, Silvia Juliana Maradei Anaya, Johanna Carolina Acosta Guio, Clara Eugenia Arteaga Diaz, Juan Carlos Prieto Rivera

**Affiliations:** 1 Maestria en Genética Humana, Facultad de Medicina, Universidad Nacional de Colombia, Bogotá, Colombia.; 2 Hospital Militar Central, Bogotá, Colombia.; 3 Instituto de Ortopedia Infantil Roosevelt, Bogotá, Colombia.; 4 Genetica Medica, Facultad de Medicina, Pontificia Universidad Javeriana. Bogotá, Colombia; 5 Hospital La Victoria, Bogotá, Colombia.

**Keywords:** Pelizaeus Merzbacher Disease, Developmental Disabilities, Child Development, Myelin Sheatn, Myelin Proteolipidic Protein, Enfermedad de Pelizaeus Merzbacher, desarrollo infantil, discapacidades del desarrollo, vaina de mielina, proteína proteolipídica de la mielina

## Abstract

**Case Presentation:**

Pelizaeus Merzbacher Disease (PMD) is an X-linked developmental defect of myelination that causes childhood chronic spastic encephalopathy. Its genetic etiology can be either a duplication (or other gene dosage alterations) or a punctual mutation at the *PLP1* locus. Clinically, it presents with developmental delay, nystagmus and, spasticity, supported by neuroimaging in which the defect of myelination is evident. We present a series of seven Colombian patients diagnosed with this leucodystrophy, describing their genotypic and phenotypic characteristics and heterogeneity.

**Clinical Findings:**

All patients included were male, 6 months to 16 years of age. Mean age at onset of symptoms was 8 months. Mean age at diagnosis was 5 years 5 months, being classic PMD most frequently diagnosed, as compared to the connatal phenotype. All cases had a primary diagnosis of developmental delay on 100%, and in 28.7% of cases, early onset nystagmus was described. 85.7% of patients had spasticity, 71.4% cerebellar signs, 57.0% hypotonia, and 28.5% had an abnormal movement disorder. Only three patients were able to achieve gait, though altered. In the two patients who had a diagnosis of connatal PMD maturational ages in danger zones according to the WHO Abbreviated Scale of Psychosocial Development were documented. All cases had abnormalities in neuroimages.

**Molecular Analysis and Results:**

Molecular studies were used in the majority of the cases to confirm the diagnosis (85.7 %). For two cases molecular confirmation was not considered necessary given their affected male brothers had already been tested. *PLP1* gene dosage alterations (duplications) were found in 28.5 % of the patients (two siblings), whereas three different single nucleotide variants were detected.

**Clinical Relevance:**

According to these findings, as authors we propose the diagnostic algorithm in Colombian population to begin on a high clinical suspicion, followed by paraclinical extension, moving on to the molecular confirmation by using approaches to simultaneously sequence the *PLP1* gene in order to detect point mutations and in/dels and performing a copy number variation analysis for the detection of gene dosage alterations.

## Resumen

**Descripción del caso:**

La enfermedad de Pelizaeus Merzbacher es una leucodistrofia ligada al X que causa encefalopatía espástica crónica en la infancia. Su etiología es genética, por duplicaciones u otros trastornos de la dosis génica o mutaciones puntuales del gen *PLP1*, lo que condiciona la formación anormal de las vainas de mielina principalmente en el sistema nervioso central. Clínicamente se caracteriza por un cuadro de retardo del neurodesarrollo, nistagmus y espasticidad, con neuroimágenes que evidencian la dismielinización. Presentamos una serie de siete casos colombianos con esta leucodistrofica en la que describimos fenotípica y genotípicamente la heterogeneidad de esta enfermedad en nuestra población.

**Hallazgos clínicos:**

Todos los pacientes analizados fueron de sexo masculino, con edad promedio de inicio de síntomas hacia los ocho meses de vida. La edad media al diagnóstico fue de 5 años 5 meses, siendo más frecuente el diagnóstico de PMD clásica que el tipo connatal. Se describe retardo del desarrollo motor en el 100% de los casos, acompañado de nistagmus en el 28.7%. 85.7% de los casos tenía algún grado de espasticidad, 71.4 % signos cerebelosos, 57.0% hipotonía, y hasta en 28.5% se evidenciaron movimientos anormales. Solo tres pacientes lograron marcha, aunque patológica. En los dos pacientes con la forma connatal se documentó una edad maduracional motora en el rango de Alerta, de acuerdo a la escala abreviada del desarrollo de la OMS. En todos los casos se detectó algún tipo de anormalidad en el estudio imagenológico cerebral.

**Estudios Moleculares y Resultados:**

El diagnóstico molecular se empleó en la mayoría de los casos (85.7%), encontrando alteraciones en la dosis génica en el 28.5% y tres diferentes mutaciones puntuales.

**Relevancia clínica:**

Dados los hallazgos en los resultados del estudio molecular, sugerimos que en el abordaje diagnóstico confirmatorio para la población colombiana se debería contemplar en un mismo tiempo tanto la secuenciación como el estudio de variantes del número de copias del gen afectado, contrario a lo sugerido en literatura mundial en la que se inicia con estudio para duplicación / deleción.

## Introduction

Pelizaeus Merzbacher Disease (PMD) is a chronic pediatric leukoencephalopathy caused by disorders of the axonal myelination and the myelin metabolism in the oligodendrocytes, reported for the first time on 1885 by doctor Friedrich Pelizaeus ^1^ and revisited on 1910 by Ludwig Merzbacher ^2^. Its genetic etiology affects the expression of the Proteolipidic Protein type 1^3^ ^,^ ^4^, varying from hemizygous mutations to gene dosage alterations of the *PLP1* (Xq22). Given the location of the causal gene, PMD is inherited in a X-linked recessive manner^3^.

Although clinical manifestations are heterogeneous ^5^ ^,^ ^6^, the most relevant neurological signs are nystagmus, developmental delay, spasticity, along with neuroimaging supporting aberrant myelination of the Central Nervous System (CNS) compromising primarily the periventricular white matter, with a tigroid striation pattern that responds to the conservation of myelinated islets, and also an alteration of the N-acetyl aspartate and choline profiles on the brain magnetic resonance spectroscopy ^5^ ^,^ ^7^. 

Unlike other leukodystrophies in which there is a period of normal cortical myelination an then comes a disruption resulting in the lost of myelin sheaths (demyelination), PMD has, from the beginning, an abnormal or low production of this very important protein (hypomyelination), due to a damage on the *PLP1* gene coding for the Protelipidic Protein type 1 that interferes with the oligodendrocyte synthesis of fully functional myelin sheaths and probably also affects the peripheral function of myelinated axons ^3^ ^,^ ^8^. 

PMD corresponds to a larger group of neurological phenotypes known as PLP1 related disorders, all being allelic diseases: Connatal PMD, Classic PMD, Nule Syndrome (NS), Complicated Hereditary Spastic Paraplegia type 2 (SPG2) and Uncomplicated Spastic Paraplegia type 2, ranging in a wide variety of clinical manifestations which variability is not yet completely understood ^5^ ^,^ ^9^. 

In general, *PLP1* gene duplications result in a classical form of PMD, nonsense mutations in either form of SPG2 and connatal form of PMD, and other monoallelic mutations have been related to less circumscribed clinical phenotypes ^6^. Patients suffering from a connatal form of PMD, the most severe phenotype, have histopathological studies revealing complete absence of myelination in the brain, explaining the rapid clinical deterioration and suggesting tan death of these patients may respond to nervous conduction alterations in brain control centers. There’s also a phenotype of patients with clinical and radiological traits almost identical to those in PMD, with no *PLP1* mutations detected, classified as PMD like (PMDL) syndrome ^9^. 

Frequently, the connatal form of PMD is expressed during the first weeks of life, through key findings in the clinical neurological examination, that include pendular nystagmus, hypotonia and laryngeal stridor; later in life, seizures and sever motor deficits appear, and hypotonia turns to weakening limb spasticity; affected patients may never walk ^10^. Verbal language is limited, but patients understand simple orders and can follow them. Affected individuals with the connatal form of PMD die in infancy, usually secondary to respiratory or deglutition complications, such as bronchoaspiration ^9^.

Classic PMD is characterized in the first stages of disease by nystagmus, hypotonia and tremor in male affected patients, joint progressively by ataxia and spastic quadriparesis in the school age. Motor impairment of the limbs is less severe tan that presented in the connatal form, and patients can frequently achieve walking even if requiring special aids, and have better control of voluntary movement of the upper limbs ^9^ ^,^ ^11^. Classic PMD affected males also have improved cognitive development, with acceptable speech. Survival rates in this patients have been described to be up to the seventh decade of life. 

On the other hand, NS patients suffer from a less harmful condition also caused by large deletions or damaging mutations resulting in loss of PLP1 protein product. As the phenotype is thought to be less severe than the other forms of PMD, some case series have even considered NS to be another variant of Complicated SPG2 ^9^. Interestingly, it has been described NS affected individuals to have a multifocal demyelinating neuropathy ^8^ ^,^ ^12^ sometimes being the only clinical feature of the syndrome; NS patients do not present with nystagmus, their spastic paraplegia is mild, affecting primarily the lower limbs, and ataxic compromise may vary. Another differential diagnosis to consider is SPG2, an allelic disorder to PMD and NS, consisting of an heterogeneous constellation of clinical phenotypes primarily characterized by weakening and progressive lower limb spasticity during the first decade of life, with previous normal motor development. Patients can also have nystagmus, optic atrophy, dysarthria, ataxic features and variable range of intellectual disability; however, symptoms appear to be less compromising tan those presenting in classic PMD. Most of mutations detected on individuals diagnosed with SPG2 are missense ^8^. Also, it is worth mentioning that SPG2 affected males can reproduce, while there are no reports of PMD affected males who have descendants ^9^.

This article describes seven Colombian individuals with clinical, paraclinical and molecular diagnosis of PMD, through phenotype and gene variant characterization. 

### Ethical approval

Written informed consent was obtained from patient’s parents / legal guardians for publication of this report. Copies of the written consents are available for review by the Editors of this journal and are kept within the clinical records of each patient.

This study was approved by the ethics committee of the Faculty of Medicine of Universidad Nacional

## Case series presentation

**Figure 1:**
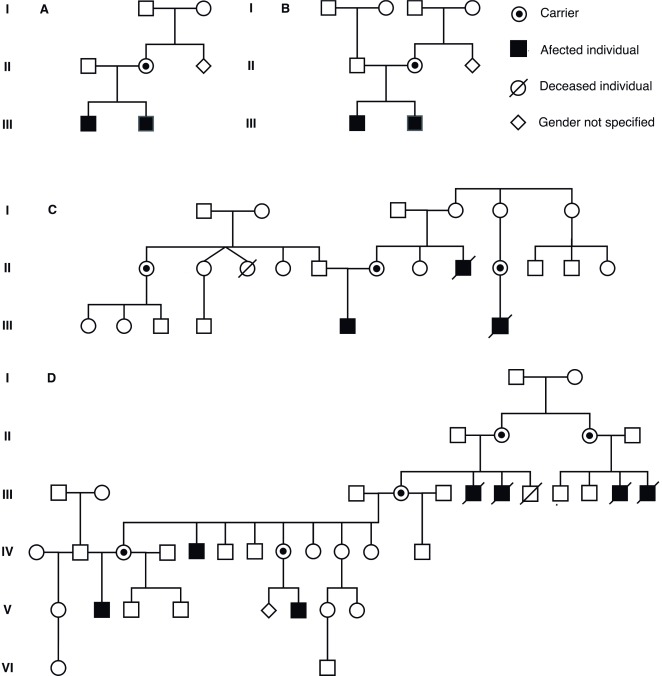
Pedigrees of families of the participant individuals. Note that patients are cited on the tables with their assigned pedigree numbers.

Seven individuals ages 6 months to 16 years (4 probands, 3 male relatives of the probands), diagnosed clinically, paraclinically and molecularly as Pelizaeus Merzbacher patients, attended in different medical care centers in Colombia (Fig. 1). They underwent clinical evaluations, neuroimaging (i.e. brain MRI), electro diagnosis (evoked visual and auditory potentials, electromyography, neural conduction velocity tests, computerized testing of gait), biochemical testing (i.e. blood and urine amino acids) and genetic testing (karyotyping, deletion/duplication analysis of *PLP1* or whole gene sequencing of *PLP1*, targeted mutation studies). Also, we applied the PMD functional disability scoring system and the WHO Abbreviated Scale of Development to assess the degree of developmental retardation and disability on our patients ^13^. 

Methodologies used for the genetic testing of *PLP1* gene were either multiplex ligation-dependent probe amplification (MLPA) using SALSA P022 A1 or B1 and P071 kits (MRC-Holland), which tested for deletions or duplications, whole gene sequencing by Sanger method for the detection of point mutations or small in/dels, or QT-PCR to confirm the presence of family mutations. In order to evaluate the impact of the molecular alterations detected, we used software as PolyPhen - 2 v.2.1 (http://genetics.bwh.harvard.edu/pph2/), HumVar model (http://genetics.bwh.harvard.edu/pph2) and SIFT (http://sift.jcvi.org) to predict pathogenic or benign changes on the PLP1 proteic product, having as reference sequence the one published under the entry NM_000533.3 (NCBI RefSeq, http://www.ncbi.nlm.nih.gov/nuccore) in the NCBI public databases. 

## Results

All patients were male, 6 months to 16 years of age, one of them died by the age of 5 due to complications of a respiratory infection. Mean age of onset of symptoms was 8 months, presenting with developmental delay on 100% of the cases and early onset nystagmus in 28.7%. Mean age at diagnosis was 5 years 5 months, being classic PMD most frequently diagnosed, in five cases, whereas the connatal phenotype was only present in two of the patients, (28.5%).

In our sample, two patients had history of cerebral palsy, being an actual comorbidity in only one of them. It is worth saying that all patients exhibited some level of speech delay or learning difficulties, and that only two were going to school. 

In the physical examination, 57.0% of the patients had horizontal nystagmus while the others had the classic rotatory phenotype; none of them had oculomotor palsy or optic nerve atrophy; three individuals had any degree of sensorineural hearing loss. 57.0% showed signs of hypotonia, 28.5% an abnormal movement disorder, 71.4% any cerebellar signs, 85.7% any degree of spasticity, annotating that only three achieved gait with evident difficulties. 

As for the two patients who had diagnosis of connatal PMD, it was documented both had experienced swallowing or deglutory disorders, history of seizures, microcephaly (in just one of them) and maturational ages in danger zones according to the WHO Abbreviated Scale of Psychosocial Development (WHO Abbreviated Scale of Psychosocial Development, https://www.unicef.org/bolivia/integrated_local_development_1480.htm).

When testing them for the *PMD functional disability scoring system*, all seven individuals had any level of disability, being moderate in 57.0% of the patients (10 to 20 points) or severe in 28.5% (under 10 points); it was not possible to evaluate the score on one patient given his very young age (Clinical endpoints shown in Table 1). 

**Table 1:** Results from the clinical evaluation of patients with Pelizaeus Merzbacher Disease.

Clinical features	1. III - 1	1. III - 2	3. III - 7	2. III - 2	2. III - 1	4. V - 2	3. III - 6
Age at onset	2 yr.	14 ms.	6 ms.	5 yr.	1 yr.	6 ms.	0 ms.
Age at diagnosis	5 yr 11 ms.	2 yr y 8 ms.	1.5 yr.	9 yr.	12 yr.	7 yr.	3 ms.
First symptoms	No head support or crawling.	DD. Nystagmus.	Hypotonia. DD.	DD.	DD.	No head support or crawling.	Laryngeal stridor. Nystagmus.
DD/ID	(+)	(+)	(+)	(+)	(+)	(+)	(+)
Nystagmus	Horizontal.	Horizontal.	Rotatory.	Rotatory.	Rotatory.	Horizontal.	Horizontal.
Language alteration	(+)	(+)	(+)	(+)	(+)	(+)	NA.
Spasticity	(+)	(+)	(+)	(+)	(+)	(+)	(-)
Walking	(-)	(-)	(-)	(+)	(+)	(+)	NA.
Swallowing issues	(-)	(-)	(+)	(-)	(-)	(-)	(+)
Hypotonia	(-)	(+)	(+)	(-)	(-)	(+)	(+)
Dysmetria/ Dysdiadochokinesia / Ataxia	(+)	(+)	(-)	(+)	(+)	(+)	(-)
PMD disability scoring system	8 PTS.	11 PTS.	2 PTS.	14 PTS.	18 PTS.	13 PTS.	NA.
WHO ASC	72 PTS.	94 PTS.	29 PTS.	115 PTS.	86 PTS.	100 PTS.	25 PTS.

Yr: Year. Ms: Months. DD: Developmental delay. ID: Intellectual disability. NA: Not applicable. PTS: Points. WHO ASC: World Healt Organization Abbreviated Scale of Development. (+): Present. (-): Absent.

Neuroimaging of patients with classic PMD showed evidence of T2 hyperintensities both diffuse or periventricular in the supratentorial withe matter. Other encephalic structures such as the brainstem, basal nuclei and cerebellum showed no abnormalities. In the connatal form affected individuals, we also observed hypo intensities of the basal nuclei and grey matter atrophy. 42.8% of the patients presented abnormal evoked auditory potentials and 28.5% abnormal evoked visual potentials; only one patient had abnormal neuro conduction velocities en another one had high levels of Mio inositol when tested for brain spectroscopic patterns. 

Molecular studies were used in the majority of the cases to confirm the diagnosis. For two cases molecular confirmation was not considered necessary given their affected male brothers had already been tested. *PLP1* gene dosage alterations (duplications) were found in 28.5% of the patients (two siblings), whereas three different single nucleotide variants were detected: c.140T>C (p.I47T), a missense variant classified as pathogenic, and two previously unreported alterations, the c.609C>T (p.Q99X) nonsense variant in two patients*,* and in one patient the c.152T>A (p.F51Y) missense variants. Laboratory endpoints are shown in Table 2. 

**Table 2:** Results from the paraclinical evaluation of patients with Pelizaeus Merzbacher Disease.

Paraclinical evaluation	1. III - 1	1. III - 2	3. III - 7	2. III - 2	2. III - 1	4. V - 2	3. III - 6
Connatal absence or myelin on MRI	(-)	(-)	(+)	(-)	(-)	(-)	(+)
MRI Hyperintensities localization	SC. PV.	Diffuse.	Diffuse.	PV.	PV / PaV.	PV. / PaV.	Diffuse.
Cerebral Spectroscopy	RP.	Normal patterns.	PV white matter irregular signal on T2.	Normal patterns.	Normal patterns.	PV posterosuperior white matter irregular signal on T2.	Normal patterns.
Auditory Evoked Potentials.	Auditory desynchrony. Type A tympanometry.	Auditory desynchrony vs. Auditory neuropathy. Severe compromise of physiological thresholds.	Normal patterns.	Normal patterns.	Normal patterns.	Normal patterns.	Normal patterns.
Visual Evoked Potentials.	Retrocorneal functional disorder.	Diffuse compromise of retinocorial pathways with axonal lost pattern.	Normal patterns.	Normal patterns.	Normal patterns.	Normal patterns.	RP.
NCV	Normal patterns.	Peroneal axonal neuropathy.	RP.	Normal patterns.	Normal patterns.	Normal patterns.	RP.
EMG	Normal patterns.	Normal patterns.	RP.	Normal patterns.	Normal patterns.	Normal patterns.	RP.
As worth spasticity index	UL 2 / IL 4.	UL 2 / IL 3.	UL / IL 4.	UL 1+ / IL 2.	UL 1+ / IL 1+.	RB 1+ / LB 1.	NA.
PLP1 molecular analysis	Complete duplication.	Complete duplication.	c.140T>C (p.I47T) hemizygous.	c.609C>T (p.Q99X) hemizygous.	c.609C>T (p.Q99X) hemizygous.	c.152T>A (p.F51Y) hemizygous.	c.140T>C (p.I47T) hemizygous.

(+): Present. (-): Absent. MRI: Cerebral magnetic resonance imaging. NA: Not applicable. SC: Subcortical. PV: Periventricular. PaV: Paraventricular. RP: Results pending. NCV: Nerve conduction velocities. EMG: Electromyography. UL: Upper Limbs. IL: Inferior limbs.

## Discussion

We present one of the first Latin-American series of patients with clinical diagnosis and molecular confirmation of Pelizaeus Merzbacher disease, being the classical form more frequent than the connatal form in the evaluated patients.

Developmental delay associated with nystagmus was key to diagnosis, both present in 100% of the cases. Along with the high clinical suspicion, supporting neuroimaging and molecular analysis permit an appropriate genetic counselling.

Connatal form of PMD is less frequent and far more severe than the classic phenotype. In our study, it is to note patients with the connatal form showed worse scores of disability (High severity scores in the PMD *Disability Scoring System*) and more pronounced developmental delay, and those continue to worsen until their deaths. 

Diagnosis can be mistaken primarily with SPG2, also caused by mutations on the *PLP1* gene, differing on signs such as autonomic dysfunction and characteristic paraplegia. NS, a variant of the PMD spectrum, presents as a periphery demyelinating neuropathy. Among other differential diagnosis we can count Krabbe disease, Canavan disease, other leukodystrophies and cerebral palsy. Connatal form of PMD is more severe than the classical form, a verifiable fact in our series, and with a reported expectancy of life lower than the first decade of life.

**Figure 2:**
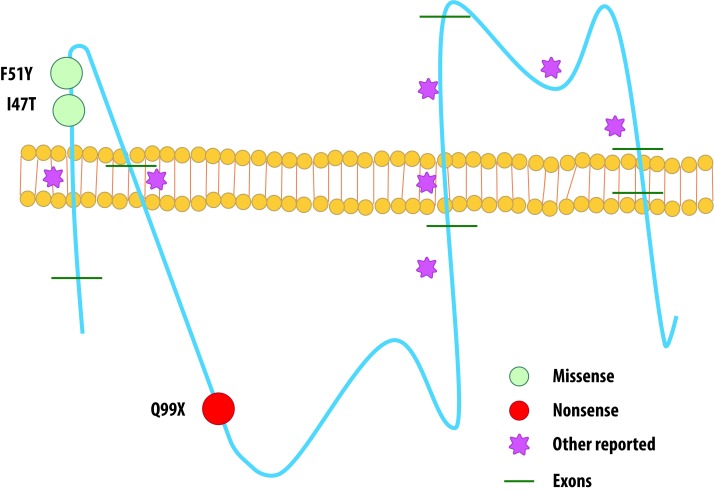
Schematic view of mutations found in our patients and previously reported mutations affecting PLP1 protein.

Point mutations were more frequently found, disregarding previous reports where >50% of *PLP1* alterations are duplications. Case series have reported point mutations as the etiology of PMD related phenotypes in nearly 30% of male affected patients, yet we report them to be present in 71.5% of our cases and cannot rule out a signature genetic background for Latin-American patients with PMD, despite simple size (Scheme of mutations found in Figure 2).

*PLP1* gene is located in chromosomal region Xq22, with a 17 kb length, 7 exons and 6 introns. Exon 1 only transcribes the start codon, while exons 2, 3, 4, and 5 encode the hydrophobic domains and the hydrophilic chains of the protein. C-terminal transmembrane domain is encoded by exons 6 and 7. Besides the C-terminal hydrophilic domain, exón 7 contains the 3’UTR ^14^ ^,^ ^15^. *PLP1* is further translated into the proteolipid protein 1 (PLP1), a 276 aminoacid peptide, or the isoform called DM20, which loses 35 residues inside its intracellular loop. Both PLP1 and DM20 are highly hydrophobic membrane proteins, accounting for up to 50% of compact myelin proteins in the central nervous system of the adult ^16^. PLP1/DM20 exact way of functioning has not been described precisely to date; however, it is clear they are needed for assembly and stability of the myelin sheath, and as before mentioned, *PLP1* mutations have been widely studied as cause of PMD and SPG2. Studying male affected patients and animal models has led us to defy PLP1/DM20 actively participate in the synthesis of myelin intraperiod line, myelin compaction, myelin sheath adhesion to oligodendrocyte membrane, etc. A wide range of mutations in *PLP1* has been described, recurrently detecting a whole gene duplication as the most frequent alteration ^17^ ^,^ ^18^ ^,^ ^19^.

Mutation c.140T>C found in our patients with the connatal form of the disease has already been reported by Hoffmann et al. in patients with classic PMD ^5^. Grossi *et al*.^20^, reported a similar mutation in a patient with a classic phenotype, an exon 2 microduplication (c.134_140dup7) that caused a frameshift (p.Ile47IlefsX4) and resulted in a truncated protein product, 4 amino acids downstream ^21^. We believe it is important to establish the biochemical functionality of I47 position on the myelin proteolipidic protein to evaluate its impact on the connatal phenotype of PMD disease, given that there are not functional studies to this date that prove in vitro or in vivo effects.

Nonsense mutation c.609C>T (p.Q99X) and missense mutation c.152T>A (p.F51Y) have not been previously reported as causes of PMD disease, but both their SIFT and PolyPhen scores suggest they are damaging (0.82 and 0.98 Polyphen scores respectively). This variants express as a compromise of two functional domains of the PLP1 protein: c.609C>T (p.Q99X) affects the cytoplasmic domain while c.152T>A (p.F51Y) affects an extracellular topological domain ^14^ ^,^ ^16^. 

In a smaller percentage of cases triplications and other dosage alterations in the *PLP1* gene have been reported, and less than 2% of cases so far reported have shown a complete or partial deletion of the gene ^22^ ^-^ ^24^. 

## Conclusions

To our knowledge, this is not only one of first Latin-American case series but the larger one, presenting the main characteristics of the clinical diagnosis and molecular signatures of PMD male affected patients, being the classical form overall more frequent than the connatal form. Both patients with the connatal form of the disease had severe disability scores and poor vital prognosis, despite having the chance of an earlier diagnosis. 

According to our results, we propose that for our population the diagnostic algorithm begins on a high clinical suspicion, followed by paraclinical extension in which neuroimaging is crucial, moving on to the molecular confirmation by using approaches to simultaneously sequence the *PLP1* gene in order to detect point mutations and in/dels and performing a deletion/duplication analysis for the detection of gene dosage alterations.

In spite of the incapacitating character of this disease, patients with less severe or moderate forms of PMD have rather normal life expectancy, but there are records of patients with severe classical forms who died past the second decade of life. Because of this, it is a priority for the clinical specialists and treating physicians to improve the diagnosis algorithms in order to shorten time before establishment of the specific therapeutic plan and the appropriate genetic counselling for the families. 
