# Supplementary material for: Clinical and mutational spectrum of Colombian patients with Pelizaeus Merzbacher Disease
Source: Colomb Med (Cali). 2018 Jun 30;49(2):182–7. doi: 10.25100/cm.v49i2.2522 (PMC6084915; doi:10.25100/cm.v49i2.2522)
Supplement: Related texts

# Case report: Heterogeneidad clínica y mutacional en pacientes colombianos con Pelizaeus Merzbacher

## Introducción

La leucodistrofia o enfermedad de Pelizaeus Merzbacher (PMD) es una encefalopatía crónica de la infancia causada por desórdenes de la mielinización axonal y del metabolismo de la mielina oligodendroglial, descrita por primera vez en 1885 por Pelizaeus ^1^ y estudiada a mayor profundidad en 1910 por Merzbacher ^2^. Su etiología es genética por mutación hemicigota o alteraciones en la dosis génica del gen *PLP1* (Xq22), que afectan la expresión de la Proteína Proteolipídica de la Mielina 1 ^3^^,^^4^. Dada la localización y función del gen causal, PMD presenta una herencia ligada al cromosoma X recesiva ^3^. 

Aunque las manifestaciones clínicas son muy heterogéneas ^5^^,^^6^, los signos clínicos primarios neurológicos incluyen nistagmo, retardo global del desarrollo, espasticidad, acompañados de características radiológicas vistas por Resonancia Nuclear Magnética cerebral (RM) como una mielinización del sistema nervioso central (SNC) aberrante de predominio periventricular sin afectación subcortical, junto a la presencia de islotes mielinizados que dan una apariencia ‘tigroide’ a la sustancia blanca, así como alteración de los patrones de N-acetil aspartato y colina en la espectroscopía que reflejan anomalías mielínicas axonales ^5^^,^^7^. 

A diferencia de otras leucodistrofias en las que la mielina se produce pero es destruida posteriormente (desmielinizantes), en los pacientes con PMD la mielina no se produce de forma adecuada. Se cree que la hipo o dismielinización responde principalmente a que la afectación de la proteína proteolipídica mielínica afectada interfiere con la formación de las vainas de mielina de los oligodendrocitos en el SNC y con la formación de éstas vainas en los axones periféricos ^3^^,^^8^.

La PMD forma parte de un grupo más amplio de fenotipos neurológicos conocidos como trastornos relacionados con el gen *PLP1*, que son todos desórdenes alélicos: la forma connatal de PMD, la forma clásica de PMD, el síndrome nulo *(SN)*, la Paraplejía Espástica tipo 2 (SPG2) complicada y la Paraplejía Espástica tipo 2 no complicada, todos ellos caracterizados por un diverso espectro de manifestaciones clínicas, cuya explicación de variabilidad no se comprende completamente ^5^^,^^9^. 

En general, se considera que las duplicaciones del gen *PLP1* dan lugar a la forma clásica de PMD, las mutaciones *nonsense* a las formas de Paraplejía Espástica complicada o no complicada (SPG2) y a la forma connatal de PMD y otras mutaciones monoalélicas se han relacionado con diferentes formas clínicas menos delimitadas ^6^. En los pacientes con la forma connatal de PMD, que es la más severa, el estudio histopatológico revela una ausencia completa de la mielinización en todo el cerebro, lo que podría explicar el rápido deterioro clínico y sugiere que la muerte, que en ocasiones es inexplicable, sea causada por alteraciones de la conducción nerviosa en los centros de control vital encefálicos. Existe un grupo de pacientes con características clínicas y neurorradiológicas casi idénticas a la PMD, en los que no se detectan mutaciones de *PLP1* y que se clasifican como pacientes con un síndrome PMD *like* (PMLD) ^9^. 

Clínicamente, la forma connatal de PMD se hace manifiesta en las primeras semanas de vida, por hallazgos al examen neurológico del recién nacido que incluyen nistagmos pendular, hipotonía, y estridor laríngeo; en edades más avanzadas aparecen crisis epilépticas y déficit motor severo y la hipotonía tiende a transformarse en espasticidad de las extremidades que puede ser muy limitante, con pacientes afectados que pueden nunca lograr la marcha o tener buena funcionalidad de sus miembros superiores ^10^. El lenguaje verbal es limitado, pero los pacientes comprenden órdenes sencillas. Los individuos afectados con esta forma de PMD mueren en la infancia, generalmente por complicaciones relacionadas con problemas deglutorios, como lo es la broncoaspiración ^9^. 

La forma clásica de PMD descrita por Pelizaeus y posteriormente por Merzbacher, se caracteriza inicialmente por la aparición de nistagmos, hipotonía y tremor en los varones afectados, que se acompañan progresivamente de ataxia y cuadriparesia espástica. Lo anterior ocurre en los primeros años de vida. El compromiso motor de las extremidades es menos severo que en la forma connatal, logrando la marcha pero usualmente con requerimiento de dispositivos de apoyo y en porcentaje importante de los casos con un adecuado control voluntario de los miembros superiores ^9^^,^^11^. Los afectados también tienen mejor desarrollo cognitivo que aquellos que padecen la forma connatal, con lenguaje verbal aceptable. La supervivencia de los pacientes con esta forma de la enfermedad se ha descrito incluso hasta la séptima década de la vida. 

Existe también una variante de PMD conocida como Síndrome Nulo (SN). Se trata de una condición causada por la presencia de grandes deleciones genómicas o mutaciones que resultan en la pérdida de las proteínas PLP1/DM20 en la que los pacientes presentan un único fenotipo clínico descrito como más leve que el causado por otras mutaciones en el mismo gen, y que en algunas series de pacientes es considerada como una forma complicada de SPG2 ^9^. De manera interesante, se describe que estos pacientes presentan una neuropatía periférica desmielinizante multifocal ^8^^,^^12^, que incluso puede ser el único hallazgo del SN; los pacientes con SN no presentan nistagmos y la paraplejia espástica que padecen es usualmente leve, se asocia a variables grados de ataxia, y afecta principalmente a los miembros inferiores. 

El segundo diagnóstico diferencial a tener en cuenta es la paraplejia espástica hereditaria de tipo 2 (SPG2), enfermedad alélica a PMD y el SN. SPG2 representa un grupo de desórdenes genéticamente heterogéneo con una amplia variabilidad clínica, en el que individuos que la padecen generalmente tienen desarrollo motor normal en los primeros años de vida, con inicio de debilidad más espasticidad progresiva de las extremidades inferiores entre los 2 y los 10 años de edad; los pacientes pueden presentar también nistagmos, atrofia óptica, disartria, ataxia, y grados variables de discapacidad intelectual en las formas complicadas; sin embargo, estos síntomas aparecen con menor severidad que en el fenotipo clásico de PMD. Las mutaciones hasta ahora descritas encontradas en individuos con diagnóstico de SPG2 son principalmente de sentido erróneo (*missense*) ^8^. Cabe anotar que se han reportado descendientes de varones con SPG2 pero no de varones con PMD ^9^. 

En este artículo se describe a siete individuos con diagnóstico clínico, paraclínico y molecular compatible con PMD a partir de la descripción fenotípica y genotípica de esta enfermedad en pacientes colombianos.

### Consideraciones éticas

Se obtuvo el consentimiento informado de los acudientes legales de los pacientes para la publicación de este reporte de casos.

Este estudio fue aprobado por el comite de etica de la Facultad de Medicina de la Universidad Nacional de Colombia

## Presentación de casos

La metodología utilizada para las pruebas genéticas del gene PLP1 fue la amplificación de sondas dependiente de ligandos múltiples (MLPZ) usando los kits SALSA P022 A1 o B1 y P071 (MRC-Holland), para probar delecion o duplicación y ña secuenciación del gen por el método de Sanger para la detección de mutaciones puntuales o in/del pequeños, o QT-PCR para confirmar la presencia de mutaciones familiares. Para evaluar el impacto de las alteraciones moleculares usamos los programas PolyPhen - 2 v.2.1 (http://genetics.bwh.harvard.edu/pph2/), HumVar model (http://genetics.bwh.harvard.edu/pph2) y SIFT (http://sift.jcvi.org) para predecir los cambios patogenicos o benignos en el produto proteico de PLP1, teniendo como referencia la secuencia de la publicación NM_000533.3 (NCBI RefSeq, http://www.ncbi.nlm.nih.gov/nuccore) en la base de datos publica de NCBI.

## Resultados

Se incluyeron 7 pacientes con edades de 6 meses a 16 años (4 probandos, dos hermanos y un primo, todos afectados) con diagnóstico clínico, paraclínico y molecular de PMD, atendidos en diferentes centros asistenciales de Colombia. Las genealogías de los casos se muestran en la Figura 1. Dependiendo del subtipo de PMD (clásico o connatal), de la historia natural de la enfermedad y de sus diagnósticos diferenciales iniciales, los pacientes tuvieron acceso a neuroimágenes (RM cerebral simple / contrastada / espectroscopía), electrodiagnóstico (potenciales evocados auditivos, potenciales evocados visuales, electromiografía, velocidades de neuroconducción o test computarizado de marcha), pruebas bioquímicas (estudios diagnósticos para descartar aminoacidopatías o acidemias orgánicas, por ejemplo) y genéticas (cariotipo, estudio molecular de gen *PLP1* y estudio de portadores), para poder determinar el diagnóstico final. Además se tuvo en cuenta las variables clínicas y paraclínicas para poder determinar el grado de severidad de la enfermedad empleando el *PMD functional disability scoring system*, el cual evalúa el impacto de la discapacidad en esta patología ^13^. 

**Figura 1:** Genealogías de los casos presentados. Se incluyeron siete pacientes con diagnóstico clínico, paraclínico y molecular de PMD, en **A**. Los individuos III.1 y III.2, en **B**. Los individuos III.1 y III.2, en **C** los casos III.6 y III.7, en **D** el individuo V.2. Se demuestran las relaciones de consanguinidad entre algunos de ellos, comentadas en el texto: Dos parejas de hermanos, una pareja de primos, y un caso en quien no se conoció a otros familiares afectados.

Para los estudios moleculares se realizaron varios abordajes técnicos, tanto para el análisis de dosis génica como para la detección de mutaciones puntuales. En el primer caso se empleó el test de amplificación múltiple de sondas dependiente de ligamiento (MLPA por sus siglas en inglés), usando el SALSA P022 A1 o B1 y P071 (MRC-Holland), de acuerdo a las recomendaciones del proveedor o el protocolo de QT PCR cuantitava, y en un segundo abordaje se empleó secuenciación del gen *PLP1*, ya que el análisis por MLPA para deleción o duplicación fue negativo. 

En el análisis de la variantes encontradas en el gen *PLP1*, se empleó la secuencia de referencia NM_000533.3 (NCBI RefSeq, https://www.ncbi.nlm.nih.gov/gene/5354) y para valorar el impacto de las mutaciones en la estructura proteica y la función se sometieron los resultados a diferentes programas de predicción bioinformática como PolyPhen - 2 v.2.1 (http://genetics.bwh.harvard.edu/pph2) y SIFT (http://sift.jcvi.org).

## Resultados

Todos los pacientes analizados fueron de sexo masculino, con edades entre 6 meses a 16 años, uno de ellos fallecido a los 5 años de vida por infección respiratoria complicada. La edad promedio de inicio de síntomas fue de 8 meses, con retardo del desarrollo motor en el 100% de los casos, acompañado de nistagmos en el 28.7%. La media de edad de diagnóstico fue de 5 años 5 meses, siendo el diagnóstico de PMD clásica el más frecuente, con cinco pacientes, mientras el tipo connatal se encontró en dos individuos relacionados (28.5%).

En esta muestra, dos pacientes presentaron como antecedente, diagnostico de parálisis cerebral, estando realmente presente como comorbilidad en uno de ellos, lo cual se pudo confirmar por los hallazgos prenatales de su historia clínica. Adicional al retardo del desarrollo existente en esta muestra, todos los pacientes cursaron también con algún grado de retardo del lenguaje. De los 5 pacientes mayores de 5 años, solamente 2 se encontraban escolarizados y su rendimiento académico fue irregular. 

Al examen físico de los pacientes descritos, el 57.0% cursaron con nistagmus horizontal y los 3 pacientes restantes con enfenotipo rotatorio clásico; ninguno presentó parálisis de oculomotores ni atrofia de nervio óptico y 3 pacientes tuvieron hipoacusia neurosensorial. El 57.0% presentaron hipotonía, el 28.5% movimientos anormales, el 71.4% signos cerebelosos, el 85.7% tenían algún grado de espasticidad. Cabe anotar que tres 3 pacientes lograron la marcha, aunque esta es fue patológica.

En los dos pacientes con la forma connatal se documentó dificultad para la alimentación, antecedentes de crisis convulsivas, en uno de ellos se encontró microcefalia y ambos tenían una edad maduracional motora en el rango de Alerta, de acuerdo a la escala abreviada del desarrollo de la OMS (WHO Abbreviated Scale of Psychosocial Development, https://www.unicef.org/bolivia/integrated_local_development_1480.htm). 

Al aplicar el *PMD functional disability scoring system* sobre los 7 pacientes, todos presentaron puntajes de discapacidad moderada (10 a 20 puntos en el 57.0% de los pacientes) a severa (por debajo de 10 puntos en el 28.5%), excepto por un paciente en el que por su corta edad no fue posible evaluarlo. Los resultados del análisis clínico se encuentran resumidos en la Tabla 1. 

**Tabla 1:** Resultados de la evaluación clínica de los pacientes con Pelizaeus Merzbacher.

Caractersísticas clínicas	1. III - 1	1. III - 2	3. III - 7	2. III - 2	2. III - 1	4. V - 2	3. III - 6
Edad de inicio	2 años	14 meses	6 meses	5 años	1 año	6 meses	0 meses
Edad al diagnóstico	5 años 11 meses	2 años y 8 meses	1.5 años	9 años	12 años	7 años	3 meses
Primeros síntomas o signos	DD	DD. Nistagmos	Hipotonía. DD	DD	DD	DD	Estridor laríngeo. Nistagmos
DD/ID	+	+	+	+	+	+	+
Nistagmos	Horizontal	Horizontal	Rotatorio	Rotatorio	Rotatorio	Horizontal	Horizontal
Trastorno del lenguaje	+	+	+	+	+	+	NA
Espasticidad	+	+	+	+	+	+	-
Marcha	-	-	-	Autónoma, con dificultad	Autónoma, con dificultad	Autónoma, con dificultad	NA
Trastornos deglutorios	-	-	+	-	-	-	+
Hipotonía	-	+	+	-	-	+	+
Dismetría/ Disdiadoconesia / Ataxia	+	+	-	+	+	+	-
PMD disability scoring system	8 PTS	11 PTS	2 PTS	14 PTS	18 PTS	13 PTS	NA
WHO ASC	72 PTS	94 PTS	29 PTS	115 PTS	86 PTS	100 PTS	25 PTS

DD: Retardo del desarrollo. ID: Discapacidad Intelectual. NA: No aplica. PTS: Puntos. WHO ASC: World Healt Organization Abbreviated Scale of Development / Escala abreviada del desarrollo de la OMS. (+): Presente. (-): Ausente.

Las neuroimagenes de los pacientes con PMD clásico reportaron hiperintensidades tanto difusas como periventriculares de la sustancia blanca supratentorial. Las estructuras como el tallo cerebral, los núcleos basales y el cerebelo no presentaron alteraciones. En los casos de la forma connatal, se observó, además de lo descrito arriba, hipointensidades de los núcleos basales y atrofia de sustancia gris. El 42.8% presentaron alteraciones en los potenciales evocados auditivos y en el 28.5% en los visuales; un paciente mostró alteraciones en la neuroconducciones y en un caso, se observó un incremento de Mioinositol determinado en la espectroscopia por resonancia. 

El diagnóstico molecular se empleó en la mayoría de los casos (85.7%), encontrando alteraciones en la dosis génica en el 28,5% y tres diferentes mutaciones puntuales, dos de ellas no reportadas previamente. Dos pacientes presentaron la duplicación completa del gen *PLP1*, otros dos presentaron la mutación misssense c.140T>C (p.I47T), en dos pacientes se encontró la mutación *nonsense* c.609C>T (p.Q99X) y otro paciente presentó la mutación missense c.152T>A (p.F51Y), estas dos últimas, privadas. Los resultados del análisis paraclínico de los casos se encuentran resumidos en la Tabla 2. 

**Tabla 2:** Resultados de la evaluación paraclínica de los pacientes con Pelizaeus Merzbacher.

Paraclínicos	1. III - 1	1. III - 2	3. III - 7	2. III - 2	2. III - 1	4. V - 2	3. III - 6
Ausencia connatal de mielina en la resonancia magnética	-	-	+	-	-	-	+
Localización de hiperintensidades en la resonancia magnética	SC. PV	Difusas	Difusas	PV	PV / PaV	PV /PaV	Difusas
Espectroscopia cerebral	SD	Patrones normales	Señal irregular de la materia blanca PV en T2	Patrones normales	Patrones normales	Señal posterosuperior PV de materia blanca irregular en T2	Patrones normales
Potenciales Evocados Auditivos	Desincronía auditiva. Timpanometría tipo A.	Desincronía auditiva vs. Neuropatía auditiva. Grave compromiso de los umbrales fisiológicos	Patrones normales	Patrones normales	Patrones normales	Patrones normales	Patrones normales
Potenciales Evocados Visuales	Trastorno funcional retrocorneal	Compromiso difuso de las vías retinianas con patrón e pérdida axonal	Patrones normales	Patrones normales	Patrones normales	Patrones normales	SD
VNC	Patrones normales	Neuropatía axonal peronea.	SD	Patrones normales	Patrones normales	Patrones normales	SD
EMG	Patrones normales	Patrones normales	SD	Patrones normales	Patrones normales	Patrones normales	SD
Índice de espasticidad de Asworth	MS 2 / MI 4	MS 2 / MI 3	MS / MI 4	MS 1+ / MI 2.	MS 1+ / MI 1+.	MS 1+ / MI 1	NA.
Estudio molecular de PLP1	Duplicación completa del gen	Duplicación completa del gen	c.140T>C (p.I47T) hemicigoto	c.609C>T (p.Q99X) hemicigoto	c.609C>T (p.Q99X) hemicigoto	c.152T>A (p.F51Y) hemicigoto	c.140T>C (p.I47T) hemicigoto

(+): Presente. (-): Ausente. RM: Resonancia Nuclear Magnética cerebral. NA: No aplica. SC: Subcortical. PV: Periventricular. PaV: Paraventricular. SD: Sin Dato. VNC: Velocidades de Neuroconducción. EMG: Electrommiografía. MS: Miembros superiores. MI: Miembros inferiores.

## Discusión

Nosotros presentamos la serie mas grande de pacientes Latinoamericanos con diagnóstico clínico y confirmación molecular del espectro de Enfermedad de Pelizaeus Merzbacher, donde la presentación más frecuente se observó en la forma clásica, sin embargo también se lograron identificar dos pacientes con forma connatal. 

Fueron claves para el diagnóstico en estos pacientes la presencia de un retardo global del desarrollo asociado a cualquier tipo de nistagmos, ambos signos estuvieron presentes en todos los pacientes. Fue necesario el diagnóstico molecular confirmatorio para el adecuado asesoramiento genético de las familias. 

La forma connatal de la PMD es menos frecuente y mucho más severa que la forma clásica. En nuestro estudio fue notable que los pacientes con la forma connatal tienen mayor discapacidad (Puntajes en rangos de alta severidad en el PMD *Disability Scoring System*) y un más pronunciado retardo en el neuro desarrollo hasta el momento en que fallecen. Cabe mencionar que si bien no alcanzan rangos de alerta en la Escala Abreviada del Desarrollo, es probable que se deba a la temprana edad en que mueren. 

En este estudio, en pacientes con diagnóstico de PMD fueron más frecuentes las mutaciones puntuales que las duplicaciones, contrario a lo reportado en literatura, donde el 50% de las alteraciones del gen *PLP1* son defectos en la dosis génica (Fig. 2). Las series de casos de la literatura reportaron la aparición de mutaciones puntuales causales en un 30% de los varones con PMD, nosotros encontramos mutaciones puntuales en el 71.5%, probablemente debido al tamaño de la muestra, aunque no se debe descartar un *background* genético particular en latinoamericanos para esta patología.

**Figura 2:** Mutaciones encontradas en nuestros pacientes y las previamente reportadas en PLP1. La figura recoge un esquema de la localización a nivel de la proteína de las mutaciones que se detectaron en los casos aquí presentados (Missense, Nonsense) vs. Las mutaciones reportadas previamente en la literatura (Otras reportadas).

El gen *PLP1* está localizado en la región cromosómica Xq22, tiene una longitud de 17 kb, contiene 7 exones y 6 intrones. El exón 1 codifica solo para una metionina y los exones 2, 3, 4, y 5 codifican los dominios hidrofóbicos y la secuencia de cadenas hidrofílicas. El dominio C terminal transmembranal es codificado por los exones 6 y 7. Este último contiene una secuencia C terminal hidrofílica y la región 3’ no trascrita ^14^^,^^15^. El gen *PLP1* Codifica para la proteína proteolipídica de mielina tipo 1 llamada también lipofilina (PLP1) de 276 aminoácidos y su isoforma generada por splicing alternativo DM20. Tanto PLP1 como DM20 son proteínas de membrana muy hidrofóbicas. La proteína DM20 pierde 35 residuos dentro de su *loop* intracelular y tanto PLP/DM20 constituyen más del 50% de las proteínas de la mielina compacta en el sistema nervioso central del adulto ^16^. Las funciones de PLP/DM20 no han sido descritas totalmente; sin embargo, es claro que son necesarias para el ensamblaje y estabilidad física de la mielina del sistema nervioso central. No sobra decir que las mutaciones en el gen *PLP1* han sido ampliamente relacionadas como causa de PMD y SPG2. Los estudios en pacientes con estas patologías, así como modelos en ratón, han permitido definir qué PLP/DM20 participa en la formación de la línea intra-período de mielina, la compactación de la mielina, la adhesión de las vainas de mielina a la membrana del oligodendrocito y el recubrimiento de mielina del axón. Un amplio tipo de mutaciones ha sido descrito en este grupo de individuos, siendo la forma más frecuentemente reportada la duplicación de gen *PLP1* ^17^^,^^18^^,^^19^.

La mutación c.140T>C que encontramos en dos de nuestros pacientes con fenotipo connatal fue recientemente reportada por Hoffman y col. pero en PMD clásica ^5^. Grossi et al.^20^,. reportaron una mutación similar en un fenotipo clásico, la microduplicación en el exon 2 de PLP (c.134_140dup7) con corrimiento en el marco en lectura (p.Ile47IlefsX4) y repercusión en una proteína truncada en 4 aminoácidos corriente abajo ^21^. Será importante en el futuro establecer la funcionalidad bioquímica de la posición Isoleucina 47 con respecto a su impacto en el fenotipo connatal, ya hasta el momento no existen estudios funcionales que permitan comprobar el efecto *in vivo* de ella. 

Las mutaciones *nonsense* c.609C>T (p.Q99X) y *missense* y c.152T>A (p.F51Y) no han sido reportadas previamente como causales, pero la predicción por SIFT y PolyPhen de su efecto en la proteína sugiere que sean probablemente deletéreas (Scores de 0.82 y 0.98 en Polyphen respectivamente). Estas mutaciones comprometen dos dominios funcionales de la proteína, en el caso de la c.609C>T (p.Q99X) se ve afectado el dominio citoplasmático y en el caso de la c.152T>A (p.F51Y) un dominio topológico extracelular ^14^^,^^16^. 

En un menor porcentaje de casos se ha reportado que pueden ocurrir triplicaciones, triplicaciones parciales e incluso quintuplicaciones del gen *PLP1*, y en menos del 2% de los casos se presentan deleciones completas o parciales de este gen ^22^^,^^23^^,^^24^. 

## Conclusiones

En nuestro conocimiento, este es el primer reporte latinoamericano de una serie de pacientes con PMD en el que se encuentra una mayor frecuencia de afectados con PMD clásica con puntajes para discapacidad moderada en quienes se detectaron más mutaciones puntuales que duplicaciones del gen *PLP1* como la causa. En los dos pacientes con la forma connatal de la enfermedad que presentamos, a pesar de realizar un diagnóstico más temprano, la discapacidad fue severa y el pronóstico vital fue siempre pobre. 

Consideramos que, en esta población, se debería contemplar un algoritmo diagnóstico que, tras la alta sospecha clínica y el soporte con neuroimagen y otros paraclínicos, confirme en simultáneo la alteración molecular de rearreglos tipo delecion / duplicación y la presencia de mutaciones puntuales en el gen *PLP1*, mediante secuenciación Sanger asociada al análisis de la dosis génica por MLPA o por otra técnica molecular disponible para ese propósito. 

A pesar de su carácter discapacitante, los pacientes con formas leves o moderadas de la Enfermedad de Pelizaeus Merzbacher tienen una esperanza de vida larga, con escasos reportes de enfermedad grave que causa el fallecimiento en la segunda década de la vida y la enfermedad en ellos progresa lentamente tras la adolescencia. Es importante mejorar el algoritmo diagnóstico para disminuir los tiempos antes del establecimiento completo de los tratamientos paliativos y para el adecuado asesoramiento genético de las familias, además que, de tratarse de la forma connatal, la muerte del paciente ocurrirá generalmente en la primera década de la vida, por lo que es importante el diagnóstico previo.
